# Early hospital readmissions after ABO- or HLA- incompatible living donor kidney transplantation

**DOI:** 10.1038/s41598-019-39841-8

**Published:** 2019-03-01

**Authors:** Juhan Lee, Deok Gie Kim, Beom Seok Kim, Myoung Soo Kim, Soon Il Kim, Yu Seun Kim, Kyu Ha Huh

**Affiliations:** 1 Department of Transplantation Surgery, Severance Hospital, Yonsei University Health System, Seoul, Republic of Korea; 2 Department of Nephrology, Severance Hospital, Yonsei University Health System, Seoul, Republic of Korea; 3 Research Institute for Transplantation, Yonsei University College of Medicine, Seoul, Republic of Korea

## Abstract

Early hospital readmission (EHR) after kidney transplantation (KT) is associated with adverse outcomes and significant healthcare costs. Despite survival benefits, ABO- and HLA-incompatible (ABOi and HLAi) KTs require desensitization and potent immunosuppression that increase risk of EHR. However, little data exist regarding EHR after incompatible KT. We defined EHR as admission for any reason within 30 days of discharge from the index hospitalization. Patients who underwent living donor KT from 2010–2017 were classified into one of three groups (control, ABOi KT, or HLAi KT). Our study included 732 patients, 96 (13.1%) of who experienced EHR. HLAi KT patients had a significantly higher incidence of EHR than other groups (26.6%; *P* < 0.001). In addition, HLAi KT (HR, 2.26; 95% CI, 1.35–3.77; *P* = 0.002) and advanced age (≥60 years) (HR, 1.93; 95% CI, 1.20–3.12; *P* = 0.007) were independent risk factors for EHR. Patients with EHR showed 1.5 times and 3 times greater risk of late hospital readmission and death-censored graft loss, respectively, and consistently exhibited inferior renal function compared to those without EHR, regardless of immunologic incompatibilities. We recommend that KT recipients experiencing EHR or its risk factors be managed with extreme care due to their increased susceptibility to adverse outcomes.

## Introduction

Kidney transplantation (KT) is the treatment of choice for end-stage renal disease patients^1^. However, thousands of patients remain on the waiting list because of ABO or HLA incompatibilities despite healthy and willing living donors^2^. These patients generally experience prolonged waiting times and are less likely to find a suitable donor than other potential recipients. Fortunately, desensitization protocols and advanced antibody detection techniques now enable successful KTs across immunologic barriers^3,4^, and although outcomes of incompatible KTs are often inferior to those of compatible KTs, these transplants offer greater survival benefit compared with remaining on dialysis^5^.

Early hospital readmission (EHR), defined as readmission within 30 days of initial discharge, increases healthcare cost and is strongly associated with morbidity^6^. Despite recent progress, EHR is common after KT^7,8^ and is associated with adverse outcomes such as late readmission, graft loss, and mortality^9–12^. Prior studies identified several risk factors for EHR including age, sex, comorbidities, and length of stay (LOS)^10,13–15^. However, each KT recipient carries distinct immunologic risks that also significantly affect the postoperative course^16^. For example, patients with ABO or HLA incompatibility (ABOi or HLAi) require potent immunosuppression, including desensitization, and are at higher risk of rejection or infectious complications after successful KT^17–19^. Therefore, immunologic status should be considered when monitoring transplant recipients.

Due to a continual lack of organs and a rapid increase of end-stage renal disease patients, occurrence of incompatible KT will inevitably rise as well^2,20^. Understanding the burdens, risks, and outcomes related of EHR in incompatible KT patients is important for their long-term care. Therefore, the goals of this study involved quantifying the incidence of EHR and evaluating its relationships with graft survival, late hospital readmission, and renal function after ABOi and HLAi KT.

## Results

### Study population

**Figure 1:**
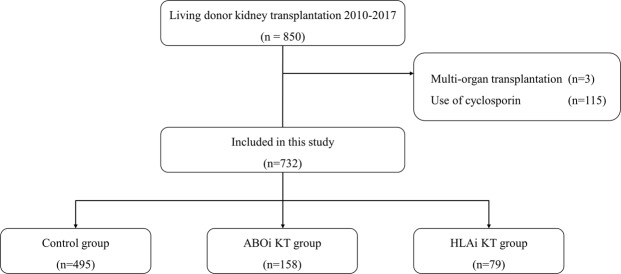
Study flow diagram.

This retrospective analysis was conducted on all consecutive living donor KT between January 2010 and December 2017 at Severance Hospital. A total of 732 patients who met the inclusion/exclusion criteria were included in the study (Fig. 1). Of these patients, 158 underwent ABOi KT and 79 underwent HLAi KT (14 combined HLAi and ABOi KT).

### Demographic and baseline clinical characteristics

Patient characteristics are summarized in Table 1. We found no significant differences in body mass index (BMI), dialysis duration, and history of diabetes among the three KT groups. However, the proportion of female recipients and re-transplant cases were significantly higher in the HLAi KT group than in the other groups. HLAi KT patients were significantly older and had higher number of HLA mismatches than those in other groups. Of HLAi KT patients, 26 (32.9%) exhibited a positive complement-dependent cytotoxicity (CDC) crossmatch, and 53 (67.1%) had a positive flow cytometric crossmatch. Patients with CDC crossmatch positive had significantly higher mean fluorescence intensity than those with flow cytometric positive (9031 ± 5912 vs. 5867 ± 4694, P = 0.013). A total of 26 patients (33%) had DSA against both class I and class II. Twenty-one recipients (26.9%) had class I DSA and 31 (39.7%) had class II DSA. Of ABOi KT patients, 122 (77.2%) had low titer of anti-A/B (≤1:64) and 36 (22.8%) had high titer of anti-A/B (≥1:128). We observed no significant differences in donor characteristics among the groups, with the exception of a lower proportion of female donors in the HLAi KT group. The use of anti-thymocyte globulin for induction was significantly more common for patients in the HLAi KT group than for those in the ABOi KT and control groups. Mean serum tacrolimus concentrations during the entire study period were similar in the three groups (P > 0.05). The median duration of the follow-up period was 45 months.

**Table 1:** Demographic and baseline clinical characteristics of participants.

	Controls (n = 495)	ABOi KT (n = 158)	HLAi KT (n = 79)	*P*-value
Female	188 (38.0%)	53 (33.5%)	63 (79.7%)	<0.001
Age (years)	46.0 ± 12.1	46.1 ± 11.3	50.1 ± 9.8	0.014
BMI (kg/m^2^)	22.7 ± 3.4	22.7 ± 3.4	22.3 ± 2.7	0.561
Re-transplant	26 (5.3%)	11 (7.0%)	17 (21.5%)	<0.001
History of diabetes	118 (23.8%)	42 (26.6%)	21 (26.6%)	0.723
History of CVD	16 (3.2%)	15 (9.5%)	7 (8.9%)	0.003
Hepatitis C virus	6 (1.2%)	2 (1.3%)	1 (1.3%)	0.955
HLA mismatch	3.2 ± 1.6	3.5 ± 1.6	3.8 ± 1.3	0.004
Dialysis duration	15.6 ± 33.5	13.6 ± 30.8	18.7 ± 27.3	0.516
Female donor	288 (58.2%)	100 (63.3%)	33 (41.8%)	0.006
Donor age (years)	42.1 ± 11.7	42.7 ± 10.8	42.4 ± 12.3	0.845
Donor BMI (kg/m^2^)	23.1 ± 2.7	23.1 ± 2.6	23.5 ± 2.4	0.492
Induction agents				<0.001
Basiliximab	488 (98.6%)	152 (96.2%)	28 (35.4%)	
ATG	7 (1.4%)	6 (3.8%)	51 (64.6%)	
Follow-up (months)	45.0 (22,71)	46.5 (21.8, 70)	42.0 (26,59)	0.376

### Index hospitalization

Clinical outcomes during index hospitalizations are shown in Table 2. During the pre-KT hospital stay, we performed thorough medical, immunological, and psychological evaluation. ABOi KT and HLAi KT patients had significantly longer pre-KT hospital stays than control group patients (*P* < 0.001), while the length of post-KT hospital stays in the HLAi KT group was significantly longer than the stay lengths of both ABOi KT and control patients (*P* < 0.001).

**Table 2:** Index hospitalization parameters.

	Controls (n = 495)	ABOi KT (n = 158)	HLAi KT (n = 79)	*P*-value
Length of stay (days)				
Pre-KT	8 [8, 8]	9 [8, 10.25]	10 [8, 14]	<0.001
Post-KT	14 [13,16]	15 [14, 17]	16 [14, 24]	<0.001
Numbers of pre-KT plasmapheresis	—	4.0 ± 2.3	4.3 ± 1.8	0.329
Delayed graft function, n (%)	17 (3.4%)	3 (1.9%)	7 (8.9%)	0.024
Biopsy-proven acute rejection, n (%)				
Antibody-mediated rejection	19* (3.8%)	7 (4.4%)	15 (19.0%)	<0.001
T-cell mediated rejection	10 (2.0%)	2 (1.3%)	1 (1.3%)	0.507
Mean eGFR at discharge	68.4 ± 20.6	68.6 ± 24.3	66.6 ± 25.9	0.785
Mean trough level of tacrolimus at discharge (ng/mL)	4.9 ± 2.1	4.5 ± 2.2	4.2 ± 2.0	0.436

*Seven cases of 19 were mixed rejection (Antibody-mediated rejection with T-cell mediated rejection).

The incidence of delayed graft function was significantly higher in the HLAi KT group than that of other groups (3.4% in controls, 1.9% in ABOi KTs, and 8.9% in HLAi KTs; *P* < 0.001). Fifty-four patients developed biopsy-proven acute rejection during their index hospitalization for KT [41 antibody-mediated rejection (AMR) and 13 T-cell medicated rejection (TCMR)]. The median time from KT to biopsy was 8 days [interquartile range (IQR), 5–14 days]. HLAi KT patients exhibited significantly higher rates of AMR than ABOi KT and control groups (19.0% in HLAi KTs, 4.4% in ABOi KTs, and 3.8% in controls, respectively; *P* < 0.001). We found no significant differences among the groups for TCMR incidence (*P* = 0.507). No patients died or experienced allograft loss during index hospitalization for KT.

### Parameters of EHR

**Figure 2:**
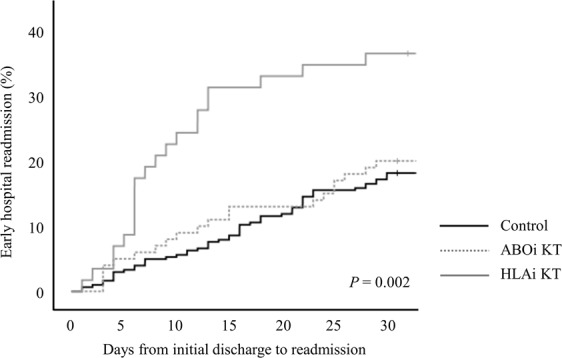
Cumulative incidence of early hospital readmission by immunologic group.

During the study period, 96 recipients experienced EHR (13.1%). As shown in Fig. 2, 55 patients (11.1%) in the control group, 20 patients (12.7%) in the ABOi KT group, and 21 patients (26.6%) in the HLAi KT group experienced EHR (*P* = 0.002). The median time from transplant discharge to EHR was 13 days (IQR, 6–22 days) with no significant differences seen among groups (*P* = 0.154). The median LOS for EHR was 8 days (IQR, 4–15 days) with a maximum of 149 days. Median LOS were similar in the control (8 days) and ABOi KT groups (7.5 days) but significantly longer in the HLAi KT group (12 days, *P* = 0.034).

The leading causes of EHR were infection (35.4%), creatinine elevation (30.2%), and surgical complication (10.4%) (Table 3). The proportion of infection was significantly higher in the HLAi KT group (52.4%) than in the other groups (30.9% in control and 30% in ABOi KT group, *P* = 0.009).

**Table 3:** Causes of early hospital readmission.

	Controls (n = 55)	ABOi KT (n = 20)	HLAi KT (n = 21)
Infection	17 (30.9%)	6 (30%)	11 (52.4%)
Creatinine elevation	15 (27.3%)	9 (45%)	5 (23.8%)
Surgical complication	7 (12.7%)	1 (5%)	2 (9.5%)
Gastrointestinal tract disorder	6 (10.9%)	1 (5%)	0
Metabolic disorder	2 (3.6%)	1 (5%)	1 (4.8%)
Urologic disorder	2 (3.6%)	0	0
Cardiologic disorder	1 (1.8%)	1 (5%)	0
Others	5 (9.1%)	1 (5%)	2 (9.5%)

Thirty-three patients underwent biopsies for acute allograft dysfunction during their EHR (n = 13, TCMR and n = 10, AMR). Most cases of rejection in the control group were diagnosed as TCMR. In contrast, AMR was found in 12.5%, 27.3%, and 83.3% of control, ABOi KT, and HLAi KT biopsied patients, respectively. There was no mixed rejection during EHR.

### Outcomes and risk factors of EHR

**Figure 3:**
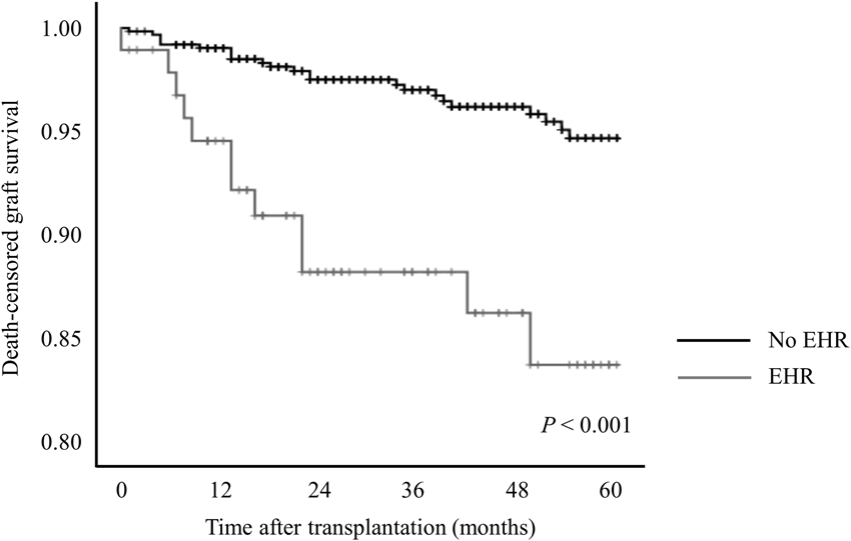
Death-censored graft survival by early hospital readmission.

During EHR, one patient died due to gastrointestinal bleeding, and another patient experienced graft loss due to refractory TCMR. As shown in Fig. 3, death-censored graft survival rates were worse for KT patients with EHR than those without EHR (86.5% *vs*. 94.8% at 5 years; *P* < 0.001) but were comparable for control, ABOi KT, and HLAi KT groups (94.0%, 91.9%, and 91.6% at 5 years, respectively, *P* = 0.601). The 5-year patient survival rates were 97.4% in patients with EHR compared to 96.1% in those without EHR (*P* = 0.342). In adjusted models, EHR was significantly associated with death-censored graft loss (HR: 2.97, 95% CI: 1.46–6.04; *P* = 0.003) but not with mortality (HR: 1.46, 95% CI: 0.32–6.70; *P* = 0.625).

**Figure 4:**
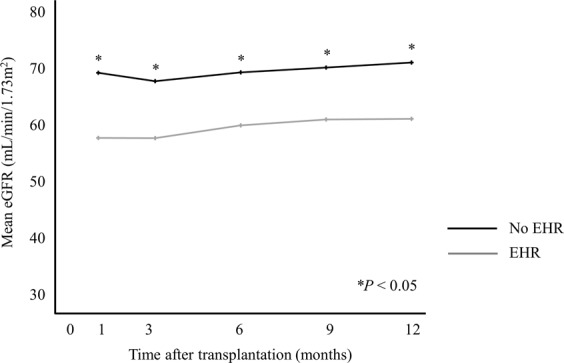
Changes in glomerular filtration rate by early hospital readmission.

Mean estimated glomerular filtration rates (eGFR) of the three KT groups were similar for 12 months after KT, while patients with EHR had consistently lower eGFR during the same period compared to those without EHR (Fig. 4).

**Figure 5:**
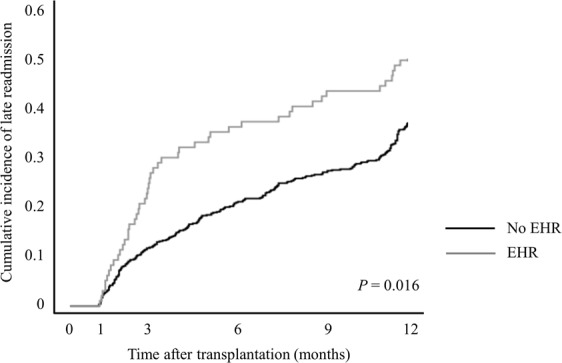
Cumulative incidence of late readmission by early hospital readmission.

Late hospital readmission occurred for 284 patients (n = 181 controls, n = 68 ABOi KTs, and n = 35 of HLAi KTs, respectively, *P* = 0.198). Of patients with EHR, 50% (48/96) were readmitted within 1 year, while only 37.1% (236/636) of those without EHR were readmitted within 1 year after transplantation (Fig. 5, *P* = 0.016). The risk of AMR during the late hospital readmission period was not significantly different among the groups (8.9% in HLAi KT, 5.7% in ABOi KT, and 4.0% in control groups, *P* = 0.162).

Cox regression analysis revealed advanced age (≥60 years) and HLAi KT as significant independent risk factors for EHR (Table 4). History of diabetes and cardiovascular disease, BMI, index hospitalization LOS, and ABOi KT status were not significantly associated with EHR.

**Table 4:** Risk factors for early hospital readmission.

Variable	Univariate		Multivariate	
	HR (95% CI)	*P*-value	HR (95% CI)	*P*-value
Female	0.891 (0.591, 1.344)	0.581		
Immunologic risk				
ABOi KT	1.123 (0.673, 1.874)	0.656	1.118 (0.670, 1.866)	0.669
HLAi KT	2.414 (1.459, 3.993)	0.001	2.258 (1.352, 3.772)	0.002
History of diabetes	1.184 (0.766, 1.831)	0.447		
History of CVD	1.218 (0.564, 2.629)	0.616		
Age ≥60 years	1.895 (1.177, 3.051)	0.009	1.933 (1.199, 3.119)	0.007
BMI ≥ 25 kg/m^2^	1.330 (0.832, 2.125)	0.233		
Delayed graft function	2.021 (0.936, 4.364)	0.073	1.723 (0.775, 3.831)	0.182
Biopsy-proven acute rejection*	1.645 (0.828, 3.267)	0.155	1.311 (0.639, 2.691)	0.460
Length of stay (post-KT)	1.004 (0.987, 1.021)	0.657		

*During index hospitalization.

## Discussion

In this study, HLAi KT significantly increases the risk of EHR compared to ABOi KT and immunologically compatible KT. Additional risk factor for EHR was advanced age. Patients who experienced EHR experienced greater risk of late hospital readmission and death-censored graft loss and exhibited consistently lower eGFR compared to those without EHR during the first post-operative year.

KT recipients are vulnerable to EHR due to the large burden of comorbidities and lifelong immunosuppression. Among them, patients with high immunologic risks (ABOi or HLAi KT) receive desensitization and potent immunosuppression. Recent multi-center study reported that HLAi KT is associated with increased risk of hospital readmission compared to compatible KT^16^. Unlike prior studies, we included ABOi KT patients in order to evaluate the impact of desensitization to EHR. Interestingly, ABOi KT had a similar risk of EHR compared to compatible KT despite desensitization therapy. However, HLAi KT was associated with increased risk of EHR. The significantly higher risk of AMR in HLAi KT is a possible explanation for this result. In addition, intensified immunosuppressive therapy for AMR may further increase the risk of infectious complications, the most common cause of EHR in HLAi KT patients^21^. Our data suggest that the risk of EHR was associated with the high immunogenicity of HLAi KT patients, rather than desensitization therapy.

The incidence of EHR in our study (13.1%) was considerably lower than that found in a large population-based United States study (31%)^7^. A recent Canadian population-based study also showed a higher EHR incidence (20.7%) than in our patients^8^; however we analyzed living donor KT patients, who typically have a lower risk for EHR. In addition, differences in healthcare systems and LOS across countries could account for variations in EHR incidence rates^22,23^. Because patients in Korea are in the hospital for approximately 2 weeks following KT, adverse events occurring during this period can be treated without affecting incidence of EHR. Long LOS is common in the Korean medical environment, which has a sufficient number of beds and a national health insurance system^24^. According to OECD reports, the average LOS for all causes in Korea is 2.1 times longer than the OECD average, 2.6 times longer than the US^25^.

Similar to prior studies, we found that advanced age is associated with EHR^7^, as elderly recipients are more likely to have comorbidities at the time of KT than younger patients^14,26^. In addition, older patients are more susceptible to infections due to progressive decline in immune function^27^. Therefore, elderly recipients should be managed especially carefully, as they are more vulnerable to adverse events requiring EHR.

Our study extended the previous work on EHR and poor outcomes including late readmission and death-censored graft loss. To our knowledge, our study was first to describe the outcomes following EHR in incompatible KT patients. McAdams-DeMarco *et al*. demonstrated that EHR was associated with late readmission, graft loss, and mortality^9^. In this study, EHR was associated with an increased risk of late readmission and death-censored graft loss but not mortality. A noteworthy finding in our study involves the impact of EHR on outcomes with respect to immunological compatibility status. In addition, this is the first study to compare renal function according to EHR. Although population-based data contain highly accurate information about admission, graft failure, and mortality, they lack granular detail about immunologic risk, biopsy results, and renal function, which are vital when determining care of KT recipients.

EHR often represents an adverse event, results in increased cost of care, and has become an indicator of healthcare quality^6^. However, emerging evidence suggests that EHR is largely driven by patient characteristics^28^. In the transplant field, immunologic risks exert a meaningful effect on risk of EHR^17^, and advanced age increases risk of readmission because of comorbidities and compromised immunity. Despite the higher risk of EHR, incompatible KT continues to demonstrate a significant survival benefit compared with remaining on dialysis^5,26^. Consequently, EHR does not necessarily result from poor quality of care, but rather represents the best possible care for these most vulnerable patients^16,29^.

Our present study has some limitations that warrant consideration. First, our single-center design limits the generalizability of this study. However, this made it possible to maintain homogeneity in the desensitization protocol and immunosuppressive regimen. Second, certain recipient risk factors previously found to be predictors of EHR (e.g., frailty, chronic obstructive pulmonary disease) were not included in our analysis, necessitating further studies to understand why these patients in particular require hospital readmission.

In conclusion, we found that HLAi KT and advanced age increase the risk of EHR after living donor KT. EHR was also associated with inferior renal function, late hospital readmission, and death-censored graft loss. Thus, KT recipients who are at risk for EHR should be managed with great care, including monitoring of DSA, renal function, and infectious complications.

## Methods

### Study population

For this retrospective single-center study, we screened 850 adult patients who underwent living donor KT between 2010 and 2017 at Severance Hospital in Seoul, Korea. Exclusion criteria included multi-organ transplantation and use of cyclosporin. Patients were classified as HLAi KT, ABOi KT, or compatible KT (no HLA or ABO incompatibility). HLAi KT patients exhibited a positive crossmatch (CDC and/or flow cytometric crossmatch) with detectable anti-HLA donor-specific antibody. Combined HLAi and ABOi transplants were classified in the HLAi KT group. Follow-up information was available for patients on death and graft loss until October 31, 2018.

### Definitions

EHR was defined as at least one readmission to the transplant hospital within 30 days after discharge from the index hospitalization^7^. The primary reason for EHR was ascertained by chart review. Late hospital readmission was defined as that occurring during the year after the EHR window^9^.

### Desensitization and immunosuppression

All ABOi or HLAi KTs were performed according to the desensitization protocol as described previously^30^. Briefly, rituximab was administered within 7 days before kidney transplantation in cases of ABOi or HLAi KT. Plasmapheresis was performed until the target antibody titer (IgG titer ≤ 1:16 in ABOi KT, negative CDC T-cell crossmatch in HLAi KT) was achieved.

The maintenance immunosuppressive regimen was tacrolimus, prednisolone, and mycophenolate mofetil (MMF). MMF was administered 1 week before transplantation in ABOi KT or HLAi KT cases. Some patients who experienced recurrent adverse events associated with MMF (gastrointestinal trouble or leukopenia) discontinued MMF at the clinician’s discretion. We administered tacrolimus 1 day before or on the day of transplantation in all patients, regardless of immunologic risk. The doses of maintenance immunosuppression were administered per institutional protocols.

### Postoperative care and renal biopsy

Before index discharge, all patients received comprehensive education and clearance from transplant team members. After discharge, patients visited the transplant clinic every week for laboratory assessments during the first post-transplant month.

Renal biopsies were performed in cases of acute allograft dysfunction (increased serum creatinine >30% from baseline or proteinuria >1 g/day). We did not perform routine protocol biopsies during the study period. All acute rejections were biopsy proven and classified into antibody-mediated rejection (AMR) or T-cell mediated rejection (TCMR) using the 2015 Banff criteria^31^. All biopsy specimens were stained for C4d.

### Prophylaxis protocol and infection monitoring

All patients received trimethoprim–sulfamethoxazole as Pneumocystis jirovecii pneumonia prophylaxis (3–6 months). Fungal prophylaxis consisted of 4 mL of oral nystatin four times daily for 3–6 months. No prophylaxis against cytomegalovirus infection was administered to any patient. Instead, a preemptive approach was used in high-risk patients (donor-seropositive and recipient-seronegative).

### Study endpoints

The primary study endpoint was the incidence of EHR. The secondary endpoints included causes and risk factors of EHR, late hospital readmission, death-censored graft survival, patient survival, and graft function. GFR was estimated using CKD-EPI (Chronic Kidney Disease Epidemiology Collaboration) equation^32^.

### Statistical analysis

Data were expressed as frequencies (percentages), means and standard deviations, or medians and interquartile ranges, as appropriate. Chi-square or Fisher’s exact tests were used to compare categorical variables. Continuous variables were compared using one‐way analysis of variance (parametric data) with *post hoc* Bonferroni analysis or Kruskal-Wallis (nonparametric data) with *post hoc* analysis with Dunn’s correction for multiple comparisons. Graft survival and EHR were analyzed using the Kaplan-Meier curves and the log-rank test. Univariate and multivariate analyses were performed using Cox proportional hazard regression models to determine risk factors for EHR. Statistical analyses were calculated using SPSS software (version 23.0; SPSS Inc., Chicago, IL, USA), and *P* < 0.05 indicated statistical significance.

### Ethics statement

All study procedures were conducted in accordance with the Declaration of Helsinki and were approved by the Institutional Review Board of Severance Hospital (4-2018-0716). Informed consent was waived by the Institutional Review Board because of the study’s retrospective design.

## Data Availability

The datasets generated during and/or analyzed during the current study are available from the corresponding author on reasonable request.
